# *Porphyromonas gingivalis*-Derived Lipopolysaccharide Promotes Glioma Cell Proliferation and Migration via Activating Akt Signaling Pathways

**DOI:** 10.3390/cells11244088

**Published:** 2022-12-16

**Authors:** Zeyuan Gao, Xiuhong Weng, Donghu Yu, Zhiyong Pan, Mingjuan Zhao, Bo Cheng, Zhiqiang Li

**Affiliations:** 1 Department of Neurosurgery, Zhongnan Hospital of Wuhan University, Wuhan 430071, China; 2 Department of Stomatology, Zhongnan Hospital of Wuhan University, Wuhan 430071, China

**Keywords:** glioma, cell proliferation, periodontitis, *Porphyromonas gingivalis*, Akt

## Abstract

Periodontitis is significantly associated with the risk of cancers in the lung and the digestive system. Emerging evidence shows a plausible link between periodontitis and several types of brain diseases. However, the association between periodontal infection and glioma remains unclear. In the cultured GL261 glioma cells, *P. gingivalis* lipopolysaccharide (LPS) significantly promoted cell proliferation at concentrations ranging from 10 to 1000 ng/mL. It promoted cell migration at a higher concentration (100 and 1000 ng/mL). Additionally, exposure to 100 ng/mL *P. gingivalis* LPS induced a significant increase in the expression of *TNF-α*, *TGF-β*, *MMP2*, and *MMP9*, as well as the phosphorylation level of Akt at Ser473. These changes induced by *P. gingivalis* LPS were significantly antagonized by the Akt inhibitor. Furthermore, a total of 48 patients with brain tumors were enrolled to investigate their periodontal status before receiving tumor management. Poor periodontal status [probing depth (PD) ≥ 6 mm and attachment loss (AL) >5 mm] was found in 42.9% (9/21) of patients with glioma, which was significantly higher than that in patients with benign tumors and the relevant data in the 4th National Oral Health Survey in China. The glioma patients with both AL > 5 mm and PD ≥ 6 mm had a higher ki-67 labeling index than those with AL ≤ 5 mm or PD < 6 mm. These findings support the association between periodontal infection and glioma progression.

## 1. Introduction

Glioma is the most common type of malignant tumor in the brain, accounting for 25.1% of the primary tumors in the central nervous system (CNS) [1]. Gliomas are classified into 4 histological grades based on the malignancy level. Lower-grade gliomas (WHO grades II and III) could evolve into glioblastoma (WHO grade IV), along with tumor progression [2]. Glioblastoma (GBM) is the most common and malignant type of glioma, with an extremely poor prognosis because of its angiogenic, aggressive, and invasive features [3]. Although the mechanism of glioma carcinogenesis is still unclear, several plausible risk factors have been investigated [4].

Chronic inflammation has been widely considered to be involved in tumor progression [5]. Periodontitis, one of the most prevalent oral diseases, is a chronic inflammatory condition characterized by the disruption of the tissues surrounding and supporting the teeth [6]. Patients with periodontal infection exhibit increased concentrations of circulating inflammatory markers, indicating the systemic implications of periodontal infection. With the recent progress in understanding the human microbiome, the association between periodontal infection and tumors has attracted significant attention [7]. Notably, individuals with periodontitis show an increased risk of total cancer compared to those with no history of periodontitis [8]. Therefore, periodontitis is significantly associated with the risk of oral, lung, pancreatic, and head and neck cancers [7].

*Porphyromonas gingivalis* (*P. gingivalis,* or *Pg*) is the most common pathogenic bacteria associated with periodontitis [9], and it plays a critical role in promoting the tumor initiation and progression when tumor patients have periodontal comorbidity. The surface biofilms of human oral squamous cell carcinoma harbor increased numbers of *P. gingivalis* compared to the neighboring uninvaded epithelial tissue [10]. Similarly, patients with gingival carcinoma had significantly increased numbers of *P. gingivalis* in tumor tissues and to a lesser extent, in the normal gingival tissues [11]. These observations suggest that *P. gingivalis* can penetrate both normal and neoplastic cells. Additionally, increased *P. gingivalis* presence was also found in pancreatic cancer [12], and orodigestive cancer [13], supporting the hypothesis that poor periodontal health is positively associated with gastrointestinal cancer risk.

Recent studies have demonstrated that periodontal disease may affect the development and progression of some brain disorders. For instance, oral *P. gingivalis* has been detected in the brains of patients with several brain diseases, such as Alzheimer’s disease and intracranial aneurysm. Likewise, periodontitis has been identified as a significant risk factor for developing higher amyloid-β plaques [14], cognitive impairment [15], and Alzheimer’s disease [16]. Furthermore, a high prevalence of periodontitis was found in patients with intracranial aneurysms, and there was an association between periodontal disease and intracranial aneurysm formation and subsequent aneurysmal subarachnoid hemorrhage [17].

Considering the association between periodontitis and tumors, we were interested in identifying whether periodontal infections are potentially linked with glioma. *P. gingivalis* LPS is commonly used to investigate how periodontal disease affects cancers [18,19,20,21,22] and CNS diseases [23,24,25]. Likewise, *P. gingivalis* LPS has been identified in brain tissue using immunofluorescence labeling [26].

In this study, we first explored the effects of *P. gingivalis* LPS on glioma cell proliferation and migration in the cultured glioma cells. In order to better determine the association between periodontal infections and gliomas, we then investigated the periodontal health status in patients with glioma. Our results showed that LPS from *P. gingivalis* could promote glioma cell proliferation and migration via activating the Akt pathway, and patients with glioma had a high prevalence of poor periodontal status.

## 2. Materials and Methods

### 2.1. Cell Culture

Mouse glioblastoma cell line GL261 was obtained from the American Type Culture Collection (ATCC, Rockville, MD, USA) and cultivated in DMEM with 10% fetal bovine serum (FBS, Gibco, Carlsbad, CA, USA) and 1% penicillin/streptomycin in an incubator at 37 °C with 5% CO_2_. The cells were subcultured every 4 days, and cells from passages 5–10 were used for experiments.

### 2.2. Cell Counting Kit-8 (CCK-8) Assay

GL261 cells were seeded (5000 cells/well) in 96-well plates and cultured for 24 h. The cells were then treated with different doses of *P. gingivalis* LPS (Invivogen, San Diego, CA, USA) dissolved in sterile water. After 24, 48, and 72 h of incubation, the cell viability was determined using a CCK-8 assay. Briefly, 10 μL of CCK-8 solution was added to each well and incubated for 4 h. The absorbance at 450 nm was assayed using a microplate reader (BioTek, Winooski, VT, USA).

### 2.3. EdU Immunofluorescence Staining Assay

GL261 cells were seeded (5000 cells/well) in 96-well plates and cultured for 24 h. The cells were then treated with or without *P. gingivalis* LPS (100 ng/mL) for 24 h. The medium was then removed, and the wells were washed using sterile PBS. The cells were incubated with EdU (10 μM, Beyotime, Haimen, China) for 2 h, and Hoechst was used to stain all the nuclei. Cell images were captured by fluorescence microscopy under ×100 magnification.

### 2.4. Wound-Healing Assay

GL261 cells were seeded into 6-well plates and grown until 90% confluence of the plate bottom was reached. A sterile pipette tip was used to make the scratch wound in each well. After a scratch, the detached cells were removed by a gentle washing, and fresh media containing different doses of *P. gingivalis* LPS was added to each well. After incubation for 24 h, cell images were collected via an inverted phase-contrast microscope (Nikon, Tokyo, Japan) and analyzed by ImageJ software (version 1.52a, National Institutes of Health, Bethesda, MD, USA).

### 2.5. Transwell Migration Assay

GL261 cells were seeded (10,000 cells/chamber) in the upper chambers in DMEM without FBS, and the bottom chambers were filled with DMEM containing 10% FBS. After 24 h, the top (non-migrated) cells were removed, and the bottom (migrated) cells were fixed and stained with crystal violet to visualize the nuclei. The number of migrating cells was counted under ×100 magnification, and the means for each chamber were measured.

### 2.6. Real-Time qPCR Analysis

The total RNA of the GL261 cells was extracted using TRIzol reagent (Thermo Fisher, Shanghai, China). The isolated RNA was reverse transcribed to cDNA using the cDNA synthesis reagent kit (Toyobo, Japan), and the expression level of the target genes was measured with SYBR Green qPCR Mixes (Bimake, Shanghai, China). The sequences of the primers are listed in Table 1. The real-time qPCR reaction was performed at 95 °C for 10 min, followed by 40 cycles of 95 °C for 15 s and 60 °C for 45 s. The expression of the target genes was calculated using the 2-ΔΔct method, and β-actin was used as the endogenous reference.

### 2.7. Western Blot

The total protein of the cells was extracted using RIPA lysis buffer, and the protein concentration was measured using a bicinchoninic acid protein assay kit (Thermo Fisher, Waltham, MA, USA). The proteins were separated using 10% SDS-PAGE gels and transferred to the PVDF membranes (Millipore, Chengdu, China). After blocking with 5% milk in Tris-buffer saline for 1 h, the membranes were incubated with primary and secondary antibodies and then visualized using enhanced chemiluminescence (ECL) reagents (VAZYME, Nanjing, China). The following primary antibodies were used: Akt (#9272, Cell Signaling Technology, Danvers, MA, USA), p-Akt (#9271, Cell Signaling Technology), and GAPDH (#8884, Cell Signaling Technology). ImageJ was used to calculate the level of target protein expression, with GAPDH as endogenous control.

### 2.8. Enrollment of Patients with Brain Tumors

Patients diagnosed with brain tumors in the Department of Neurosurgery at Zhongnan Hospital of Wuhan University were recruited between August 2020 and November 2020. The clinical data of 51 patients who underwent oral examinations after hospitalization were collected. One patient with a career history of ionizing radiation exposure and two patients with insufficient oral data were excluded from the study. Finally, 48 patients who received the oral examination were eventually enrolled in the study. The mean ages of patients with glioma and benign tumors were 56.2 and 55.1 years old, respectively. To investigate the prevalence of periodontitis in patients with brain tumors, data on periodontal health status collected in the present study were compared to those in the age-matched population from the 4th National Oral Health Survey in China [27], a cross-sectional oral health survey conducted in all 31 provinces, autonomous regions, and municipalities in China from September 2015 to June 2016.

The study was approved by the Ethics Committee of Zhongnan Hospital of Wuhan University (NO. 2021043), and all patients were informed in detail concerning the study.

### 2.9. Clinical Examination for Periodontal Health Status

Periodontal examinations were conducted using a manual periodontal probe (Hu-Friedy, Chicago, IL, USA) with 1 mm accuracy. Periodontal health status was recorded according to the probing depth (PD) of periodontal pockets and attachment loss (AL) in each permanent tooth from 6 points, according to the 4th National Oral Health Survey-Examination methods [27]. All oral examinations were performed by the same dental specialist—who had received sufficient training—before the initiation of the study.

### 2.10. Statistical Analysis

The data were expressed as mean ± standard deviation and analyzed using an unpaired *t*-test, a one-way ANOVA test, a two-way ANOVA test, or a Chi-squared test using Graphpad Prism 8.0. Data were considered statistically significant when the *p* value was < 0.05.

## 3. Results

### 3.1. P. gingivalis LPS Promotes the Proliferation and Migration of GL261 Cells

**Figure 1:**
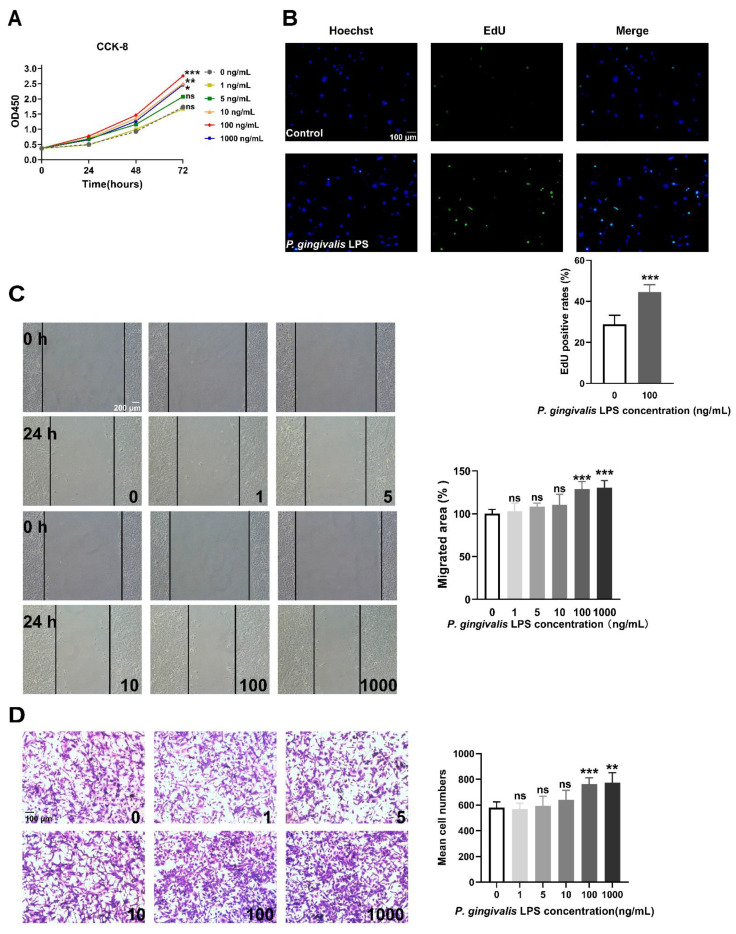
LPS derived from *P. gingivalis* promoted the proliferation and migration of GL261 glioma cells. (**A**) *P. gingivalis* LPS promoted cell viability at concentrations ranging from 10 to 1000 ng/mL (two-way ANOVA, *n* = 5). (**B**) *P. gingivalis* LPS (100 ng/mL) increased the rate of EdU positive cells (unpaired *t*-test, *n* = 6). (**C**,**D**) *P. gingivalis* LPS promoted cell migration at concentrations ranging from 100 to 1000 ng/mL according to the wound-healing assay (one-way ANOVA, *n* = 6) and the transwell migration assay (one-way ANOVA, *n* = 5). ns: no significance; * *p* < 0.05; ** *p* < 0.01; *** *p* < 0.001 vs. untreated cells (0 ng/mL).

LPS secreted from *P. gingivalis* is the most common periodontal pathogen [9]. We examined the effects of *P. gingivalis* LPS on the proliferation and migration of GL261 glioma cells. As shown in Figure 1A, *P. gingivalis* LPS at doses ranging from 10 to 1000 ng/mL significantly promoted GL261 cell viability. Moreover, the EdU assay indicated that *P. gingivalis* LPS (100 ng/mL) could promote GL261 cell proliferation (Figure 1B). The wound-healing assay and transwell migration assay indicated that a high dose (100–1000 ng/mL) of *P. gingivalis* LPS promoted GL261 cell migration, while a low dose (1–10 ng/mL) had no significant effect on cell migration (Figure 1C,D). Therefore, 100 ng/mL of *P. gingivalis* LPS was used for further experiments.

### 3.2. P. gingivalis LPS Activates the Akt Signaling Pathway and Induces Cytokine Secretion of GL261 Cells

**Figure 2:**
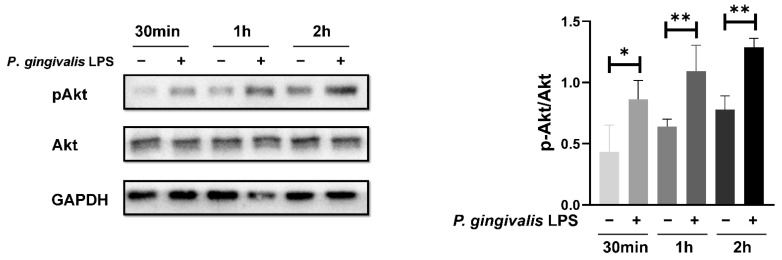
*P. gingivalis* LPS treatment induced an increase in Akt phosphorylation. * *p* < 0.05; ** *p* < 0.01; one-way ANOVA, *n* = 4.

The Akt signaling pathway is frequently activated and plays a pro-tumor role in glioblastoma. Therefore, we investigated whether the Akt pathway is involved in the response of GL261 cells to *P. gingivalis* LPS stimulation. As shown in Figure 2, exposure to 100 ng/mL *P. gingivalis* LPS induced a significant increase in the phosphorylation level of Akt at Ser473, which indicates the activation of Akt.

**Figure 3:**
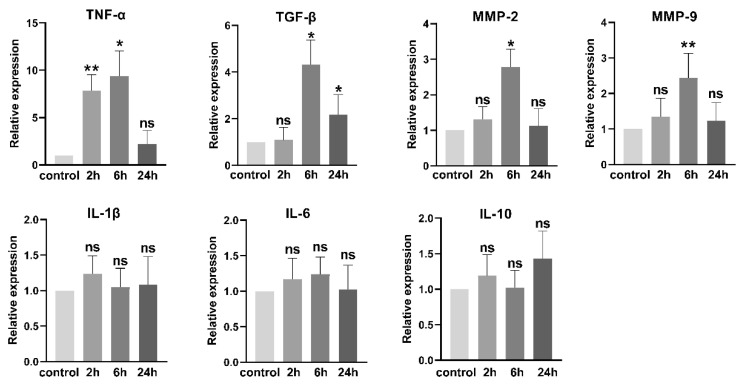
*P. gingivalis* LPS (100 ng/mL) treatment increased the mRNA levels of inflammatory cytokines and invasion-related MMPs. ns: no significance; * *p* < 0.05; ** *p* < 0.01; one-way ANOVA, *n* = 6.

We also examined the *P. gingivalis* LPS-induced changes in mRNA levels of several tumor-associated cytokines and the invasion-related matrix metalloproteinases (MMPs). As shown in Figure 3, following exposure to *P. gingivalis* LPS, the mRNA levels of *TNF-α* and *TGF-β* were significantly increased, while those of *IL-1β*, *IL-6*, and *IL-10* remained unchanged. The mRNA levels of *MMP-2* and *MMP-9* were elevated at 6 h of exposure to *P. gingivalis* LPS compared to those in the control.

### 3.3. Inhibition of Akt Activity Antagonizes P. gingivalis LPS-Induced Cell Proliferation and Migration

**Figure 4:**
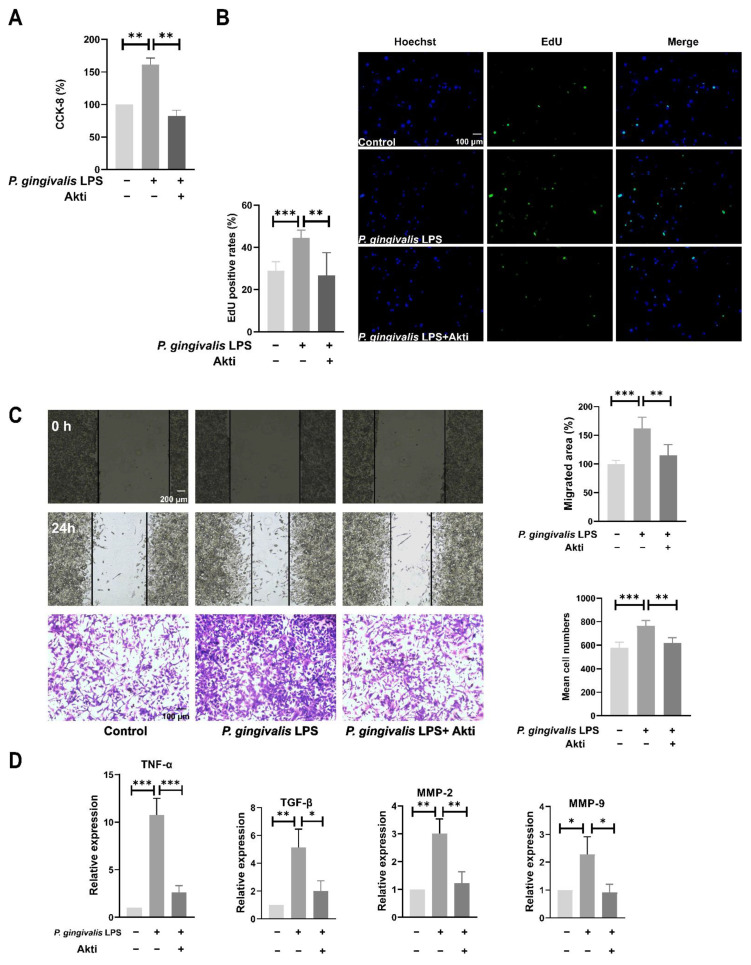
Inhibition of Akt activity antagonized *P. gingivalis* LPS-induced cell proliferation (**A**,**B**), migration (**C**), and expression of inflammatory cytokines and MMPs (**D**). GL261 cells were pretreated with 0.5μM AKT inhibitor IV for 1 h, then treated with 100 ng/mL *P. gingivalis* LPS for 6 h (mRNA assay) or 24 h (proliferation and migration assay). * *p* < 0.05; ** *p* < 0.01; *** *p* < 0.001; one-way ANOVA, *n* = 5.

To further confirm the involvement of the Akt pathway in the pro-tumor effects of *P. gingivalis* LPS, the Akt activity in the GL261 cells was inhibited by the Akt inhibitor IV (0.5 μM). Pretreatment with Akt inhibitor IV significantly antagonized *P. gingivalis* LPS-induced cell proliferation and migration (Figure 4A–C). Likewise, Akt inhibitor IV pretreatment dramatically diminished the *P. gingivalis* LPS-induced increase in mRNA levels of *TNF-α*, *TGF-β*, *MMP-2*, and *MMP-9* (Figure 4D).

### 3.4. P. gingivalis LPS Alters DNMTs and TETs Expression in GL261 Cells

**Figure 5:**
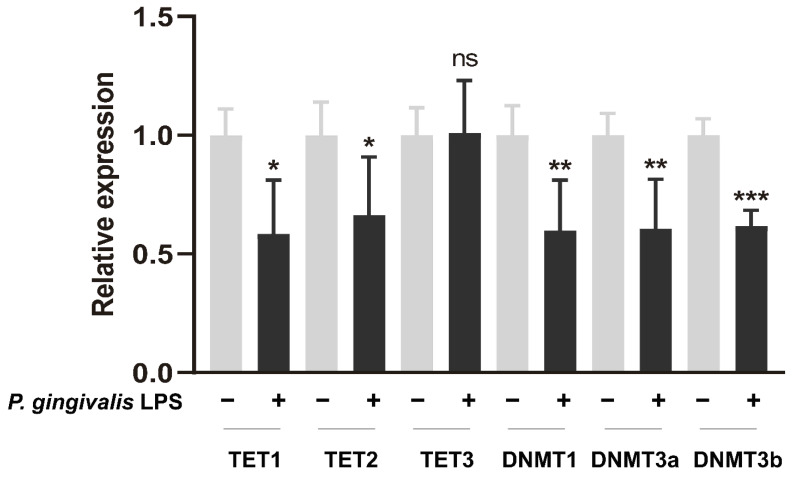
*P. gingivalis* LPS (100 ng/mL) modulated the gene expression of epigenetic enzymes in GL261 cells after exposure for 24 h. * *p* < 0.05; ** *p* < 0.01; *** *p* < 0.001; unpaired *t*-test, *n* = 5.

Epigenetic modulations are regarded as a profoundly supplementary mechanisms determining the way in which chronic inflammatory diseases affect multiple systemic disorders [28]. Previous studies showed that *P. gingivalis* LPS could alter the gene expression of the DNA methyltransferase family (DNMTs) and ten-eleven translocation proteins (TETs) in vitro [29,30,31]. Here, we investigated the mRNA expression levels of the two common epigenetic enzymes. As shown in Figure 5, the mRNA levels of *DNMTs*, *TET1*, and *TET2* were significantly attenuated after exposure to *P. gingivalis* LPS for 24 h.

### 3.5. Glioma Patients Have a Higher Prevalence of Poor Periodontal Status

**Figure 6:**
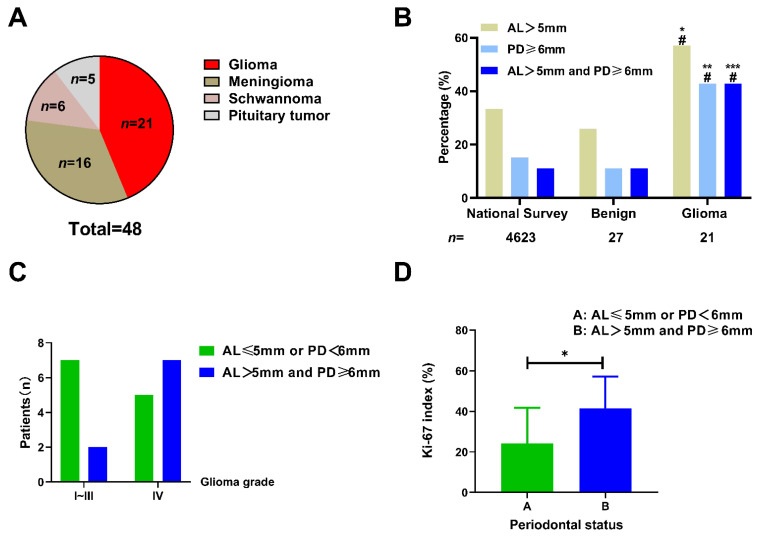
The association between poor periodontal status and glioma. (**A**) The proportion of different brain tumors exhibited by the patients enrolled in the study. (**B**) The patients with glioma had a higher prevalence of poor periodontal status than those with benign tumors or those from the Chinese population in the 4th national survey. * *p* < 0.05; ** *p* < 0.01; *** *p* < 0.001 vs. national survey; # *p* < 0.05 vs. benign tumors, Chi-squared test). (**C**) The patients with WHO IV grade glioma tended to exhibit poorer periodontal condition (the presence of both AL > 5 mm and PD ≥ 6 mm) compared with those with lower tumor grades (58.3% vs. 22.2%, *p* = 0.1842, Chi-squared test); (**D**) The glioma patients with poorer periodontal condition (the presence of both AL > 5 mm and PD ≥ 6 mm) exhibited a higher Ki-67 labeling index than those with better periodontal condition (41.43% vs. 24.25%, *p* = 0.0449, unpaired *t*-test); * *p* < 0.05.

A total of 48 patients, including 21 cases diagnosed with glioma and 27 cases with benign brain tumors (16 meningioma, 6 schwannoma, and 5 pituitary tumors) were enrolled in this study (Figure 6A). The clinical characteristics of these patients is displayed in Table 2 and Table 3. Probing depth (PD) and attachment loss (AL) are two main and essential markers indicating periodontal health status. According to the 4th National Oral Health Survey in China [27], the prevalence of AL > 5 mm and PD ≥ 6 mm in the age-matched Chinese population was 33.4% (1544/4623) and 15.1% (698/4623), respectively. The patients with benign brain tumors showed a comparable proportion of AL > 5 mm (7/27, 25.9%, *p* = 0.5401) and PD ≥ 6 mm (3/27, 11.1%, *p* = 0.7880). In contrast, those with gliomas exhibited a significantly higher proportion of AL > 5 mm (12/21, 57.1%, *p* = 0.0340) and PD ≥ 6 mm (9/21, 42.9%, *p* = 0.0022) in comparison to the population in the survey (Figure 6B and Table 2). Similarly, patients with benign tumors had a comparable incidence of both AL > 5 mm and PD ≥ 6 mm (3/27, 11.1%, *p* > 0.9999). Those with gliomas had a higher incidence (9/21, 42.9%, *p* = 0.0002) compared to the population in the survey (513/4623, 11.1%). These results demonstrated that patients with gliomas rather than benign brain tumors had a higher prevalence of poor periodontal status.

**Figure 7:**
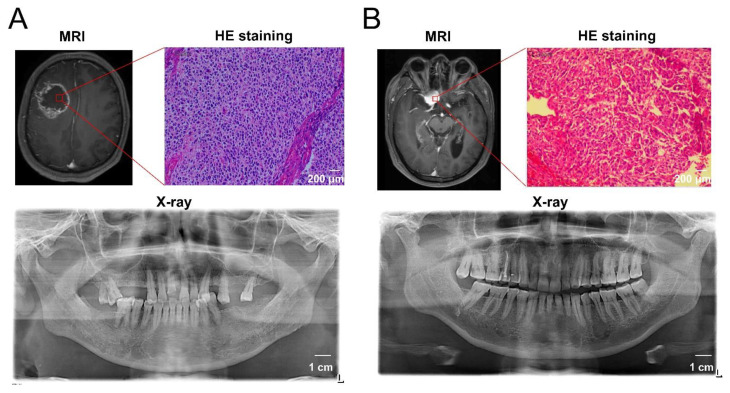
The representative MRI, histological staining, and oral X-ray images of patients with GBM and benign tumors. (**A**) A 56-year-old male patient diagnosed with glioblastoma showed a 6 mm pocket depth maximum, a 7 mm attachment loss maximum, multiple tooth loss by oral examination, and deep alveolar bone resorption by X-ray examination, indicating the comorbidity of severe periodontitis. (**B**) A 63-year-old male patient diagnosed with meningioma showed better periodontal health according to the periodontal examination and X-ray images.

Next, we investigated whether poor periodontal conditions were associated with glioma malignancy. As shown in Figure 6C, high-grade glioma (WHO grade IV) patients had a slightly higher prevalence of both AL > 5 mm and PD ≥ 6 mm compared to that of patients with lower grade gliomas (58.3% vs. 22.2%, *p* = 0.1842). Ki-67, a nuclear protein expressed in the proliferating cells, is widely considered to be a tumor proliferation marker, and a high Ki-67 labeling index generally indicates a poor clinical prognosis [32]. Of the patients with gliomas, 19 cases underwent Ki-67 histopathological examination. We further explored the correlation between the Ki-67 labeling index (the fraction of Ki-67-positive tumor cells) and the periodontal conditions in patients with gliomas. We found that patients with both AL > 5 mm and PD ≥ 6 mm had a higher ki-67 labeling index than those with AL≤ 5 mm or PD < 6 mm (41.43% vs. 24.25%, *p* = 0.0449, Figure 6D). This result indicates that glioma cells in patients with poor periodontal status exhibit a higher proliferation capacity. The representative MRI, histological staining, and oral X-ray images of patients with GBM and benign tumors are shown in Figure 7.

## 4. Discussion

Periodontitis has been linked with various systemic diseases, such as cardiovascular disease, diabetes, and multiple cancers [33,34]. Since oral *P. gingivalis* has been detected in the brains of patients with several brain diseases, the potential correlation between periodontitis and brain disorders has become a hot research topic [35]. A high prevalence of periodontitis has been identified in patients with several types of brain diseases. A case-control study that included 70 patients with intracranial aneurysms demonstrated that 49% of cases exhibited comorbidity with severe periodontitis [17]. Patients with Down’s syndrome and bipolar disorders also have an increased risk of suffering from periodontitis [36,37]. In the present study, we found that the patients with glioma had a higher prevalence of poor periodontal condition (the presence of both AL > 5 mm and PD ≥ 6 mm) than those with benign tumors and the aged-matched Chinese population from the national survey. This comorbidity link suggests that periodontitis likely influences glioma development and progression.

Periodontitis is a chronic inflammatory disease characterized as a subgingival complex microbial pathogen infection. These pathogens can penetrate periodontal tissues into the blood vessels, causing transient bacteremia through toothbrushing and dental procedures [26,38]. Several studies have identified the presence of oral bacteria such as *Porphyromonas gingivalis* in the vessel wall of intracranial aneurysm specimens [39,40]. The presence of *P. gingivalis* LPS has also been identified in the postmortem brain specimens of AD patients using immunolabeling and immunoblotting [26]. Additionally, the DNA of *P. gingivalis* was detected in the cerebrospinal fluid of AD patients [16]. Therefore, these results confirm that *P. gingivalis*, or at least its LPS, can invade the brain tissue via the blood–brain barrier [26]. Considering the comorbidity of periodontitis and glioma, we speculate that oral *P. gingivalis* infection influences glioma development and progression. We observed that a low concentration (≥10 ng/mL) of *P. gingivalis* LPS significantly promoted the proliferation of GL261 cells, whereas only a higher concentration (≥100 ng/mL) promoted the migration of GL261 cells.

LPS from *P. gingivalis* exerts its effects on various cells by stimulating cytokine secretion [41,42,43,44]. Therefore, we also investigated *P. gingivalis* LPS-induced changes in the production of inflammation and tumor-associated cytokines. We found that exposure to *P. gingivalis* LPS significantly increased the mRNA levels of *TNF-α*, *TGF-β*, *MMP-2*, and *MMP-9* and had no significant effect on the mRNA levels of *IL-1β*, *IL-6*, and *IL-10*. TNF-α can promote glioma invasion and angiogenesis. TGF-β facilitates glioma growth and immunosuppression and stimulates glioma cell migration and angiogenesis [45]. Furthermore, MMPs are of great importance in facilitating cancer cell migration and invasion by modulating the degradation of the basement membranes and the extracellular matrix. In support of our results, *P. gingivalis* LPS has been shown to upregulate the expression of MMP2 and MMP9 in oral cancer cells [46]. The increased expression of TNF-α, TGF-β, MMP-2, and MMP-9 might explain how the *P. gingivalis* LPS exerts pro-tumor effects. Notably, the Akt signaling pathway has been widely demonstrated to take part in the regulation of inflammatory processes and tumor growth. Previous studies showed that *P. gingivalis* LPS promoted cell growth and cytokine secretion via activating the Akt/NF-κB pathway [46,47,48]. In the present study, treatment with 100 ng/mL *P. gingivalis* LPS significantly activated Akt, while Akt inhibitor IV attenuated the pro-tumor effects of *P. gingivalis* LPS. These results indicate that LPS exerts the pro-tumor effects via the Akt pathway. However, further studies are necessary to investigate how *P. gingivalis* LPS activates Akt and whether other signaling pathways are involved.

Oral pathogens and their virulence are reported to generate epigenetic modulations, thus triggering inflammation [29]. In the present study, *P. gingivalis* LPS attenuated the gene expression of *DNMTs*, enzymes facilitating and maintaining the DNA methylation. The modulation of *DNMTs* corroborates with the previous findings [29,31]. The decreased expression of DNMTs may lead a hypomethylation pattern in *P. gingivalis* LPS-treated cells, while hypomethylation of the genes is closely linked to transcriptional upregulation [49], indicating that DNMTs might be involved in the cytokine upregulations in *P. gingivalis* LPS-treated cells. Furthermore, the downregulation of *TET1* and *TET2* following exposure to *P. gingivalis* LPS was observed. TETs play a crucial role in erasing the aberrant DNA methylation [28], while the aggregation of aberrant DNA methylation was known to promote cancerization [50,51]. Therefore, the decreased TETs tended to indicate a facilitation for cancer development. However, the epigenetic alterations in cancer cells are complex, and the regional hypermethylation (aberrant DNA methylation) coexists with global hypomethylation [52,53]. Hence, the impact of epigenetic modulation reacting to infectious agents needs to be deeply investigated.

Growing evidence has been emerging in recent years concerning the potential role of gut microbiota in the development and function of CNS. This interaction is termed the gut–brain axis [54]. The oral cavity, full of diverse microbes, is anatomically closer to the cerebrum and shares nearly the same vascular system. It is reasonable to speculate that oral microbial infection may also affect brain disorders. However, few studies have focused on this topic, and most have focused on the correlation between oral microbiota and Alzheimer’s disease. Whether oral microbiota plays a role in glioma progression remains unclear. However, a case-control study reported that the oral microbiota composition and gene functions are significantly associated with human brain glioma grade [55]. In support of this, our investigation indicates that LPS derived from oral *P. gingivalis* exhibits pro-tumor effects on glioma progression.

To date, the causal factors of glioma remain unknown. The low five-year survival rate disappoints the patients and their relatives. Therefore, identifying the potential causes or risk factors is pressing. Periodontitis is a common and reversible disease, and intervention of periodontal infection may benefit the prognosis of patients with glioma.

## 5. Conclusions

Periodontal infection with *P. gingivalis* shows pro-tumor effects on glioma progression via LPS-induced Akt activation. Additionally, patients with glioma have a higher prevalence of exacerbated periodontal conditions. Therefore, further understanding of this microbe–brain tumor link may lead to novel strategies for managing gliomas.

## Figures and Tables

**Table 1:** RT-qPCR primers.

	Forward	Reverse
*iL-1β*	CTTTGAAGAAGAGCCCATCC	CACTTGTTGGTTGATATTCTGTC
*iL-6*	ACAACTCATCTCATTCTGC	GTGTCCTAACGCTCATAC
*iL-10*	GGTTGCCAAGCCTTATCGGA	GGGGAGAAATCGATGACAGC
*TNF-α*	CCTCACACTCAGATCATC	AACCTGGGAGTAGACAAG
*MMP-2*	CCTGCTTAAAGGTGGCTATG	TGCCGAGGTAGAGGAAAG
*MMP-9*	GGCGTGTCTGGAGATTCG	CCTCATGGTCCACCTTGTTC
*TGF-β*	GAACCAAGGAGACGGAATAC	CCATGAGGAGCAGGAAGG
*TET1*	GAGCCTGTTCCTCGATGTGG	CAAACCCACCTGAGGCTGTT
*TET2*	CCTGGTGAACAAAGTCAGAATGGC	AATTACCATCACCGCCGTCTCACA
*TET3*	TCCGGATTGAGAAGGTCATC	CCAGGCCAGGATCAAGATAA
*DNMT1*	AAGAATGGTGTTGTCTACCGAC	CATCCAGGTTGCTCCCCTTG
*DNMT3a*	GAGGGAACTGAGACCCCAC	CTGGAAGGTGAGTCTTGGCA
*DNMT3b*	CGTTAATGGGAACTTCAGTGACC	CTGCGTGTAATRTCAGAAGGCT
*β-actin*	TGAAGATCAAGATCATTGCTCCTC	CCTGCTTGCTGATCCACATC

**Table 2:** Characteristics of brain tumor patients in our study.

	Benign Tumors (*n* = 27)	Glioma (*n* = 21)	*p*-Value
Mean age (years old) *	55.1 ± 8.9	56.2 ± 8.7	0.6851
Female/male	16/11	12/9	>0.9999
Alcohol consumption	4	2	0.6830
Smoking	8	6	>0.9999
BMI > 25	12	6	0.3693
Diabetes mellitus	3	3	>0.9999
Family history of brain tumors	1	0	>0.9999
Teeth brushing (times per day *)	1.333 ± 0.620	1.381 ± 0.590	0.7887

* The data of mean age and teeth brushing times per day were calculated by the unpaired *t*-test; the others were calculated by the Chi-squared test.

**Table 3:** The data of periodontal and pathological examinations.

Patient (No.)	Age (y o)	Gender	AL_max_ (mm) *	PD_max_ (mm)	Histologic Type	WHO Grade	Ki-67 (%)
01	45	Female	4	5	Meningioma	1	4
02	56	Female	5	5	Meningioma	1	4
03	60	Male	5	4	Meningioma	1	0
04	63	Male	2	4	Meningioma	1	3
05	43	Female	5	4	Meningioma	1	0
06	53	Female	4	4	Meningioma	1	3
07	57	Female	2	3	Meningioma	1	0
08	77	Male	5	3	Meningioma	1	0
09	48	Female	4	5	Meningioma	1	2
10	62	Female	6	3	Meningioma	1	1
11	63	Female	6	7	Meningioma	1	2
12	52	Male	4	5	Meningioma	1	3
13	39	Male	3	3	Meningioma	1	5
14	65	Male	7	4	Meningioma	2	25
15	50	Male	4	4	Meningioma	2	10
16	66	Female	5	3	Meningioma	2	7
17	53	Female	2	3	Pituitary tumor	1	2
18	60	Female	6	4	Pituitary tumor	1	1
19	43	Male	7	6	Pituitary tumor	1	3
20	56	Female	2	4	Pituitary tumor	1	2
21	54	Female	13	9	Pituitary tumor	1	1
22	46	Female	2	4	Schwannoma	1	0
23	55	Male	7	4	Schwannoma	1	0
24	47	Female	4	5	Schwannoma	1	0
25	50	Male	1	3	Schwannoma	1	0
26	57	Male	4	3	Schwannoma	1	0
27	69	Female	4	3	Schwannoma	1	0
28	39	Female	4	3	Ganglioglioma	1	1
29	54	Male	6	4	Astrocytoma	2	25
30	45	Female	3	4	Astrocytoma	2	4
31	60	Female	4	4	Astrocytoma	2	1
32	46	Female	6	6	Anaplastic Oligodendroglioma	3	50
33	58	Female	7	6	Anaplastic Oligodendroglioma	3	20
34	73	Female	5	4	Anaplastic Oligodendroglioma	3	30
35	68	Male	5	5	Anaplastic Astrocytoma	3	10
36	56	Female	4	5	Glioblastoma	3	50
37	60	Female	6	5	Glioblastoma	4	15
38	55	Female	3	5	Glioblastoma	4	40
39	56	Female	5	3	Glioblastoma	4	40
40	49	Male	6	4	Glioblastoma	4	40
41	51	Male	7	6	Glioblastoma	4	NA **
42	61	Female	7	8	Glioblastoma	4	40
43	70	Male	6	6	Glioblastoma	4	60
44	55	Male	4	4	Glioblastoma	4	35
45	46	Male	6	6	Glioblastoma	4	50
46	67	Female	6	8	Glioblastoma	4	50
47	56	Male	7	6	Glioblastoma	4	NA
48	55	Male	8	7	Glioblastoma	4	20

* The values of ALmax and PDmax referred to the maximum of PD and AL of all teeth from one patient. ** The specific Ki-67 indexes of two glioma patients were not available.

## Data Availability

Not applicable.
